# How to use intensive care unit scoring systems: a practical guide for the intensivist

**DOI:** 10.62675/2965-2774.20250347

**Published:** 2025-03-14

**Authors:** Giulliana Martines Moralez, Filipe Sousa Amado, Gloria Adriana Rocha Martins, Antonio Paulo Nassar, Jorge Ibrain Figueira Salluh

**Affiliations:** 1 Department of Critical Care Postgraduate Program in Translational Medicine Instituto D’Or de Pesquisa e Ensino Rio de Janeiro RJ Brazil Department of Critical Care and Postgraduate Program in Translational Medicine, Instituto D’Or de Pesquisa e Ensino - Rio de Janeiro (RJ), Brazil.

## INTRODUCTION

For more than 40 years, clinicians and researchers have used intensive care unit (ICU) scoring systems. These tools have been extensively tested and applied to assess the severity of acute illnesses, estimate the mean mortality, and evaluate ICU performance. Although the Acute Physiology and Chronic Health Evaluation (APACHE IV) and Simplified Acute Physiology Score (SAPS 3) are essential tools for ICU management, region-specific models have recently been developed and implemented to provide more precise predictions.^1-3^In the present study, we focused on how intensivists should use general scoring systems to assess ICU performance and how this information can guide management.

### What are intensive care unit predictive scores?

**Figure 1:**
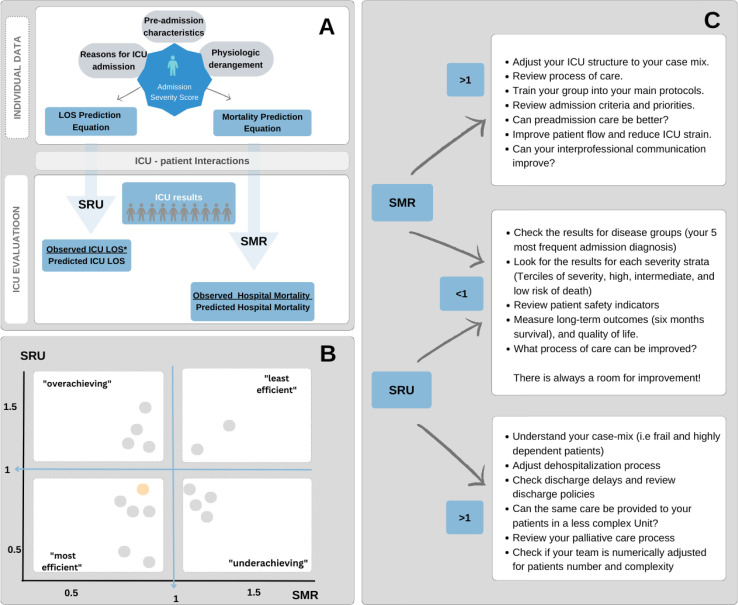
Intensive care unit scores: performance evaluation and quality management.

Intensive care unit scoring systems, such as the APACHE and SAPS, are not disease specific; they reflect the overall severity of disease in critically ill patients at ICU admission and predict the risk of in-hospital mortality.^4^These scores consider chronic health conditions, physiological and laboratory variables, reasons for ICU admission and the use of supportive therapies. These variables, when applied in an equation, provide a number that represents the severity of critical illness at baseline. Subsequent equations estimate the risk of hospital mortality and the ICU length of stay (LOS) (Figure 1A).^5^

(A) Quality metrics: from patient data to intensive care unit data; (B) Benchmarking. Rothen et al.^8^ evaluated intensive care unit efficiency using the severity-adjusted SAPS 3 mortality score and resource use combined. On the basis of the median standardized mortality rate and standardized resource use, each intensive care unit was assigned to one of four groups: “most efficient”, “least efficient”, “overachieving”, and “underachieving”.^8,10^ Each plot illustrates one intensive care unit, and it is possible to compare intensive care unit performance; (C) Improvement. We suggest organizational interventions according to each quadrant in which the intensive care unit is classified.

ICU - intensive care unit; LOS - length of stay; SRU - standardized resource use; SMR - standardized mortality rate.

### How can intensive care unit score results be interpreted?

Intensive care unit scores do not discriminate well between patients who live and those who die on an individual basis.^6^However, they do provide a mortality risk estimate. Thus, ICU managers can calculate the standardized mortality rate (SMR) by dividing the “mean observed mortality” by the “mean estimated mortality” for all patients admitted to an ICU during a given period.

When the actual hospital mortality rate is higher than the predicted mortality rate, the SMR is > 1. When the actual rate is lower than the predicted rate, the SMR is < 1. Suppose that during a specific year, 35% of all admitted patients died before hospital discharge, but the predicted mortality rate was 30%. In this case, the ICU SMR was 1.17 (i.e., 0.35/0.30). Thus, an SMR of 1.17 indicates an excess mortality rate of 17%, reflecting a potential imbalance between the quality of care and patients’ predicted conditions and outcomes.

Similarly, ICU managers can assess the standardized LOS according to severity, dividing the average observed ICU LOS by the expected ICU LOS.^7^The SAPS 3 group called this metric standardized resource use (SRU) and considered only the ICU LOS of survivors.^8^The same rationale described for the SMR can be applied here, where an SRU > 1 implies that more resources are used than are predicted by patient severity. The SRU estimates the average amount of resources (ICU days) used per surviving patient and is a well-validated proxy for hospital cost and efficiency (Figure 1B).^9^

Variations in organizational features across different ICUs can explain discrepancies between the predicted and obtained outcomes. These risk-adjusted metrics provide a data-driven approach to identify areas for improvement, implement changes, and measure their effectiveness. This continuous quality improvement cycle ensures optimal patient care within the ICU (Figure 1C).^10^

Pitfalls in intensive care unit prognostic score use (and how to overcome them):

1. Individual mortality prediction: ICU scoring systems should be used to predict mortality in a population, not in individuals. We should interpret an “x% predicted mortality” as follows: “for every 100 patients with the same characteristics, x% will probably die”.
2. Mortality prediction to guide patient care: the ICU score can inform managers but is not an adequate tool to guide goals of care or ICU interventions.
3. ICU allocation: scores are not tools for triage. ICU admission should always be guided by a clinical judgment and a prioritization framework.^11^
4. Timing: a premature analysis may not encompass all the outcomes for admitted patients. For example, if results from the first trimester are reported on April 1st, there are likely patients admitted in the last week of the first quarter who are still in the hospital. Therefore, we recommend allowing longer periods before definitive analysis and conclusions can be drawn.
5. “Transfer and discharge bias”: variation in the SMR could be partially explained by ICU and hospital discharge patterns. Every transferred patient is considered alive at discharge. Intensive care units that deal with frequent out-of-hospital transfers can artificially decrease the SMR, leading to biased comprehension of ICU quality.^12^ Considering these transfer rates allows a critical appraisal of this point.
6. “Garbage in, garbage out”: data collection is a crucial step of ICU management. Missing data and inconsistencies associated with data collection compromise calculated scores and their predictions.^13^
7. An extremely low SMR does not mean that the quality of care is good for all patients: the SMR represents an average value and fails to capture the potential variability in care and outcomes across different groups.
8. The distribution of illness severity can impact performance metrics: in ICUs with greater proportion of lower-risk patients, the scoring system may (globally) overestimate the mortality rate, a situation known as Simpson’s paradox.^14^

How can benchmarking be used for intensive care unit management?

- The ICU performance metrics of a recent period (month, trimester, year) can be presented. Context should always be provided if appropriate, such as changes in structure, process, and case mix that could be associated with the outcomes (Table 1S - Supplementary Material)
- Trends should be analyzed over time. Performance evaluation is a dynamic process. Observing how the performance metrics of ICUs change over time (instead of checking an isolated time interval) is strongly recommended.
- Comparisons of ICU performance to that of recognized benchmarks and general ICUs should be performed. In addition, comparisons with similar profiles, especially for specialized ICUs (e.g., neurological, surgical), may be relevant. Finally, the case mix should always be considered to allow for an adequate comparison.^15^

Benchmarking SMRs and lengths of stay provides ICU and hospital managers with a broader view of quality improvement. Other domains suitable for benchmarking include adherence to care processes, patient safety, economic outcomes, and patient or family satisfaction. Currently, benchmarking in real time by using electronic multinational platforms such as Epimed,^1^ANZICS,^2^and NICE is possible.^3^By leveraging real-time data, ICU managers can identify areas for improvement, implement targeted interventions, and continuously monitor intervention effectiveness.

## Conclusions

A pragmatic and rigorous interpretation of SMRs and risk-adjusted lengths of stay allows data-driven approaches to assess and improve ICU performance. These are essential principles for intensivists, as prognostic scores are valuable tools for continuous ICU evaluation and management. Understanding the benefits and limitations of these tools may ensure broader implementation and drive better organizational interventions to improve ICU quality and efficiency.

## Supplemental Material

How to use intensive care unit scoring systems: a practical guide for the intensivist


