# Supplementary material for: How to use intensive care unit scoring systems: a practical guide for the intensivist
Source: Crit Care Sci. 2025 Mar 14;37:e20250347. doi: 10.62675/2965-2774.20250347 (PMC11991817; doi:10.62675/2965-2774.20250347)
Supplement: Supplementary file 1 [file 2965-2774-ccsci-37-e20250347-suppl.pdf]

Critical Care Science
https://doi.org/10.62675/2965-2774.20250347

SUPPLEMENTARY MATERIAL

How to use intensive care unit scoring systems: a practical guide for the intensivist

Giulliana Martines Moralez[1], Filipe Sousa Amado[1], Gloria Adriana Rocha Martins[1], Antonio Paulo Nassar Junior[1], Jorge Ibrain Figueira Salluh[1]

Table 1S - Understanding the factors behind performance indicators

SMR and SRU reflect global ICU performance, but do not capture the whole picture. To analyze these indicators, a broad scenario must be provided, considering several aspects of the care, such as:	
Case mix	Case mix is the patients' characteristics of that specified ICU. For example, some subgroups of patients, who can be managed in specialized ICUs, may exhibit mortality rates overestimated by general prediction models. Age and comorbidities may vary over time and among different ICUs representing an additional challenge to accomplish optimal lengths of stay. Distribution of severity score strata can also affect performance metrics.
Structure	The presence of step-down units can result in earlier discharge and lower ICU LOS, similarly, better staffing patterns and access to specialized equipment can all influence patient outcomes.
Process	Implementation of new practices and changes in the process of care (such as admission and discharge criteria, assessment for palliative care, and infection control protocols), can significantly influence patients' outcomes.
Healthcare system	A mismatch between ICU supply and demand can disrupt an ICU ability to provide timely, high-quality care to all critically ill patients. The situation can lead to suboptimal care quality, increasing the risk of adverse events, premature discharges, unplanned readmissions, and avoidable deaths. Immediate and temporary "stress" experienced by and ICU due to capacity strain can be described by many indicators, as ICU bed occupancy and availability, time delay in patient ICU admission, refusal rate.
Additional outcome measures	Other outcome measures can provide valuable insights into care effectiveness and performance of ICU scoring systems. These quality indicators include readmission rates, unplanned reintubation rates, post discharge functional status, healthcare costs, and patient and family satisfaction.

SMR - standardized mortality rate; SRU - standardized resource use; ICU - intensive care unit; LOS - length of stay.

This is an open access article under the CC BY license (https://creativecommons.org/licenses/by/4.0/).
